# Supplementary material for: Induction of anergic or regulatory tumor-specific CD4+ T cells in the tumor-draining lymph node
Source: Nat Commun. 2018 May 29;9:2113. doi: 10.1038/s41467-018-04524-x (PMC5974295; doi:10.1038/s41467-018-04524-x)
Supplement: Supplementary file 1 — Supplementary Information [file 41467_2018_4524_MOESM1_ESM.pdf]

<u>Supplementary Information</u>

Induction of anergic or regulatory tumor-specific $CD4^+$ T cells in the tumor-draining lymph node

Alonso et al.

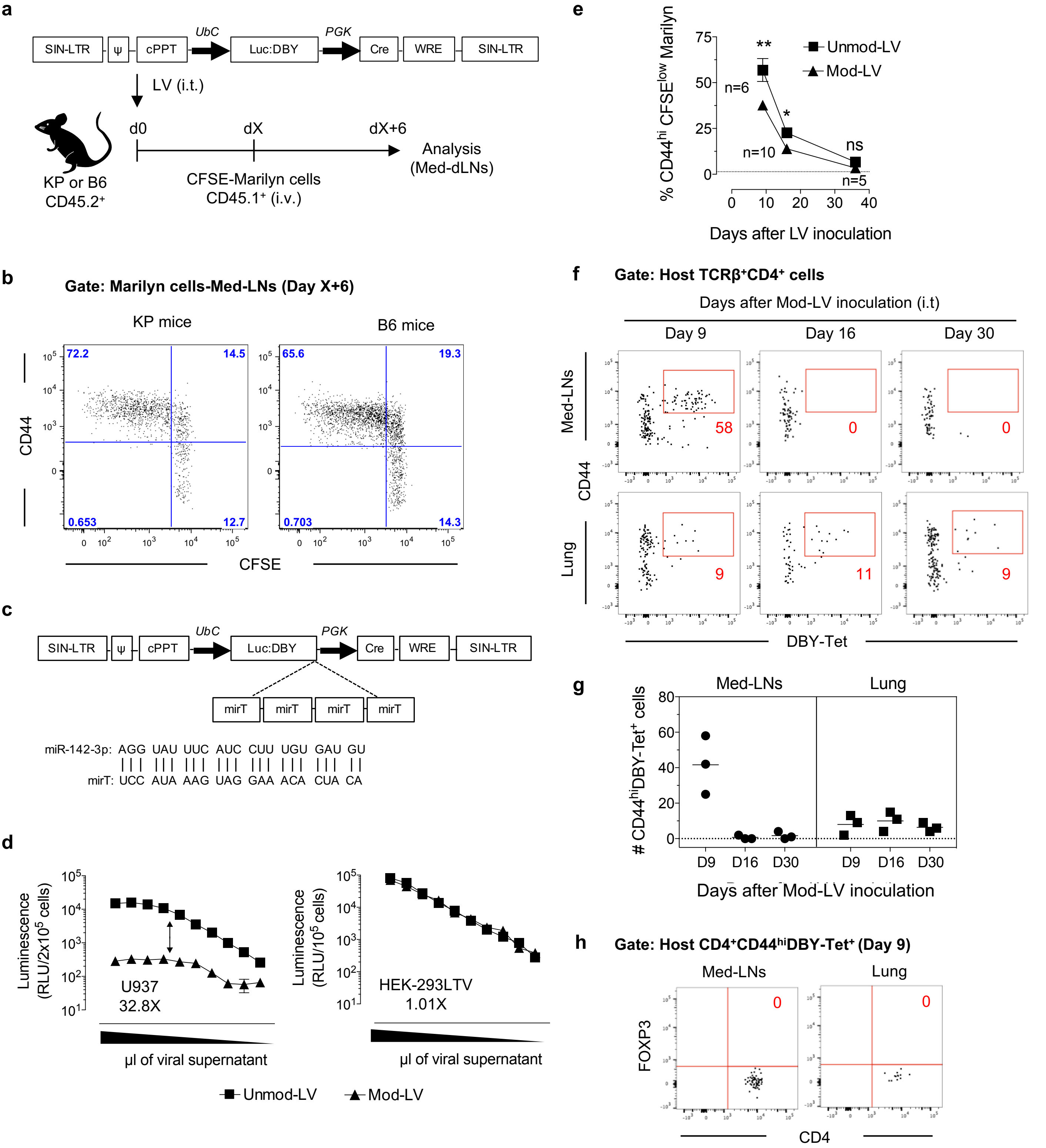

Supplementary Figure 1: Development of a genetically engineered mouse model of lung adenocarcinoma expressing a MHC-II-restricted cytoplasmic Ag in tumor cells. **(a)** Map of the Cre-encoding LV expressing a MHC-II-restricted epitope (DBY) as a fusion protein coupled to luciferase (Luc:DBY) (SIN-LTR: self-inactivating long terminal repeat, Ψ: HIV packaging signal, cPPT: central polypurine tract, PGK: phosphoglycerate kinase promoter, WRE: woodchuck post-transcriptional regulatory element, UbC: ubiquitin C promoter). The LV was inoculated i.t. into KP or B6 mice and CFSE-labeled naïve Marilyn cells were transferred i.v. 3 days later. **(b)** Activation and proliferation of Marilyn cells 6 days after transfer in the Med-dLNs. **(c)** Map of the new LV vector used to avoid DBY presentation by transduced hematopoietic cells. **(d)** Luciferase activity as a readout of DBY expression was determined in cellular lysates from epithelial (HEK-293LTV) or hematopoietic derived (U937) cell lines transduced with serial dilutions of unmodified (Unmod) or modified (Mod)-LV. **(e)** Decreased priming of naïve Marilyn cells by the Mod-LV. B6 mice were inoculated with the Unmod-LV or Mod-LV. CFSE-labeled naïve Marilyn cells were transferred after 3, 10 or 30 days and the Med-dLNs were analyzed 6 days later. Dashed line: Marilyn T cell activation in the absence of Ag. Representative of one out of three independent experiments (mean ± SEM, ns: non-significant, $*p<0.05$, $**p<0.01$ Mann-Whitney *U* test. **(f)**. Representative plots of the endogenous $CD44^{hi}$DBY:I-A^b-specific $CD4^+$ T cells after DBY:I-A^b tetramer (Tet)-based cell enrichment in B6 mice receving Mod-LV i.t. Total numbers are shown. **(g)** Quantification (number). Each point represents a pool of 2 mice. **(h)** FOXP3 expression by endogenous $CD44^{hi}$DBY:I-A^b-specific $CD4^+$ T cells 9 days after Mod-LV inoculation. One experiment out of two is shown.

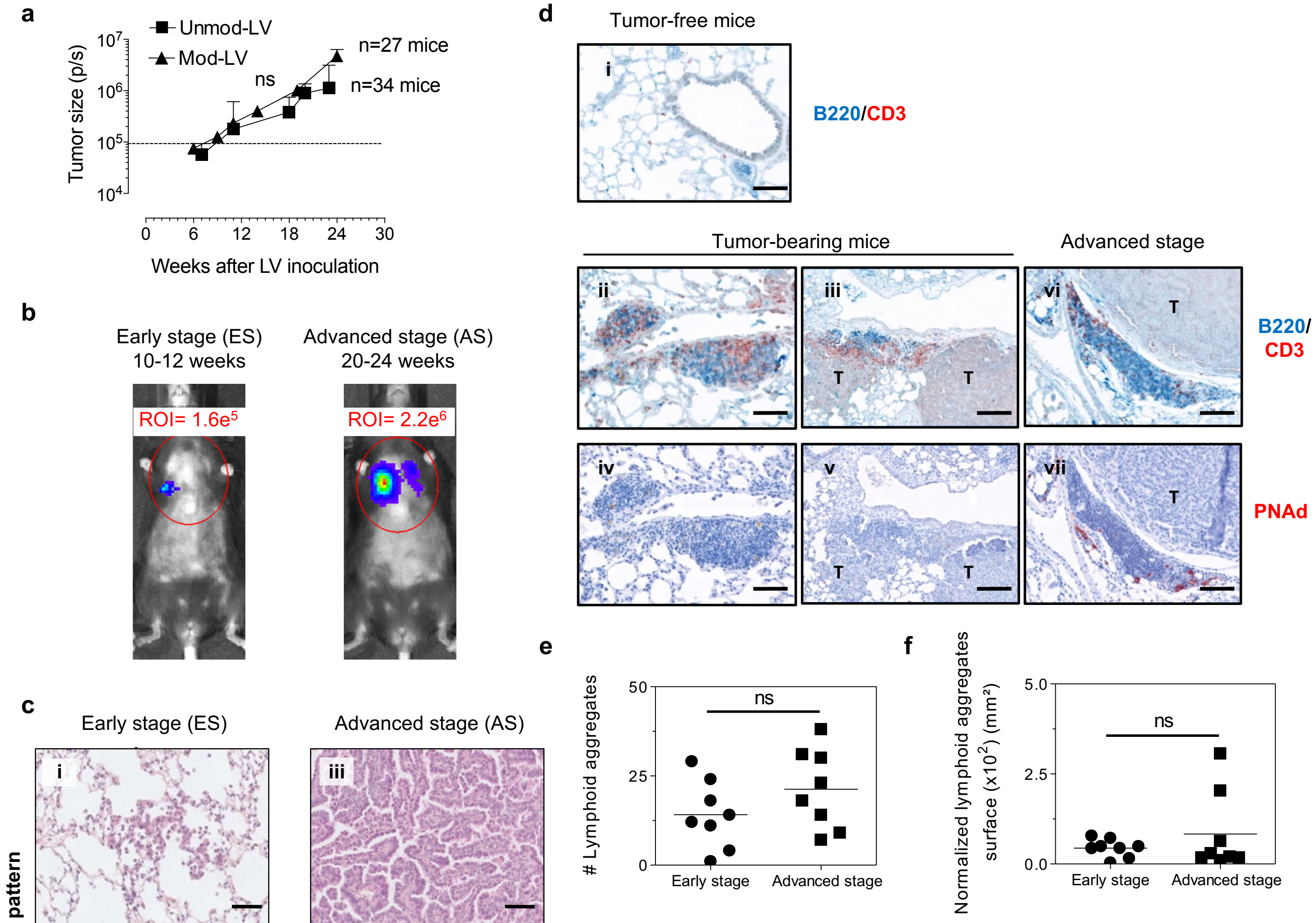

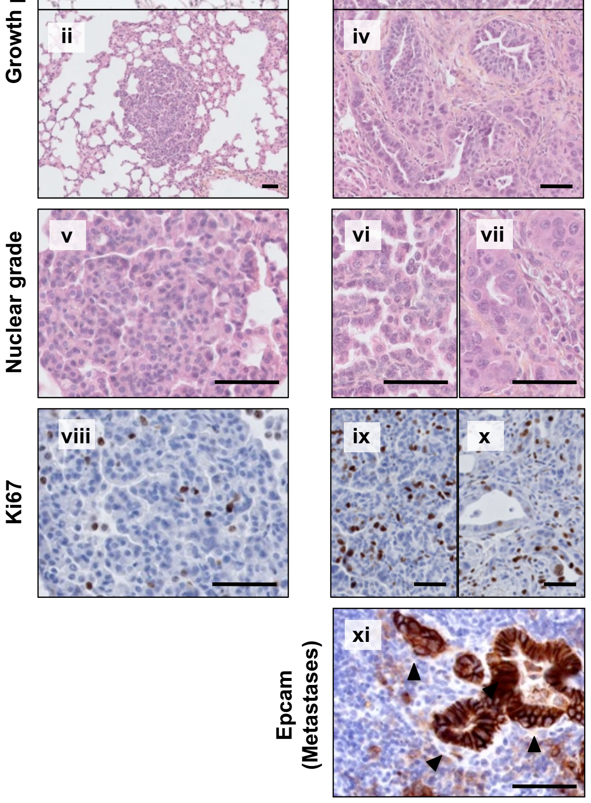

Supplementary Figure 2: Definition of early and advanced tumor stages in a genetically engineered model of lung adenocarcinoma. (a) Kinetics of tumor growth in KP mice inoculated with Unmod-LV or Mod-LV represented as total flux measurement in photons per second (p/s). One representative out of 3 experiments (geo-mean ± SEM). **(b)** Luciferase activity at early (ES) or advanced (AS) tumor stages. **(c)** Histopathological and immunohistochemical analysis of tumors from KP mice at early (n=8) and advanced (n=8) stages. **c-i-vii**: Hematoxylin/Eosin staining, **c-viii-x**: Ki67 staining and **c-xi**: Epcam staining in the Med-TdLN. **(c-i)** Premalignant lesions (Atypical adenomatous hyperplasia) and **(c-ii)** small micropapillary adenocarcinomas typically observed at ES. **(c-iii)** Papillary adenocarcinoma and **(c-iv)** acinar/glandular adenocarcinoma, the latter is associated with a well-developed desmoplastic stroma observed at AS. **(c-v)** Slightly irregular nuclei are found at ES **(c-vi, vii)** while very irregular nuclei are present at AS. **(c-viii)** Low proliferation in ES contrasting with high proliferation by **(c-ix)** papillary and **(c-x)** acinar/glandular adenocarcinoma in AS. **(c-xi)** Adenocarcinomatous acinar/glandular structures (arrow heads) detected by Epcam expression are found in the TdLN at AS. Panels **c-i**, **iii**, **iv**, **ix** and **x** are at x200 magnification. Panel **c-ii** is at x100. Panels **c-v-viii** and **c-xi** are at x400. Scale bar= 50 µm. **(d)** CD3, B220 and PNAd lung staining of tumor-free and tumor-bearing mice. **(d-ii, iii)** Lymphoid aggregates in the lung of tumor-bearing mice are associated to vascular, bronchial or tumor structures. **(d-iv, v)** Absent of PNAd expression in the intrapulmonary lymphoid aggregates. **(d-vi)** In advanced tumor stage, the lymphoid aggregates found in the adenocarcinoma invading the chest wall **(d-vii)** display PNAd expression. **(e)** Number of lymphoid aggregates containing 60 or more CD3$^+$ T cells and B220$^+$ B cells at early (n=8) and advanced stage (n=8) in the whole lung of tumor-bearing mice. **(f)** Normalized lymphoid aggregate surface corresponds to the ratio of total lymphoid aggregate surface/total lung surface. All panels are at x200 magnification. Scale bar= 50 µm. T=tumor. ns: non-significant Mann-Whitney *U* test.

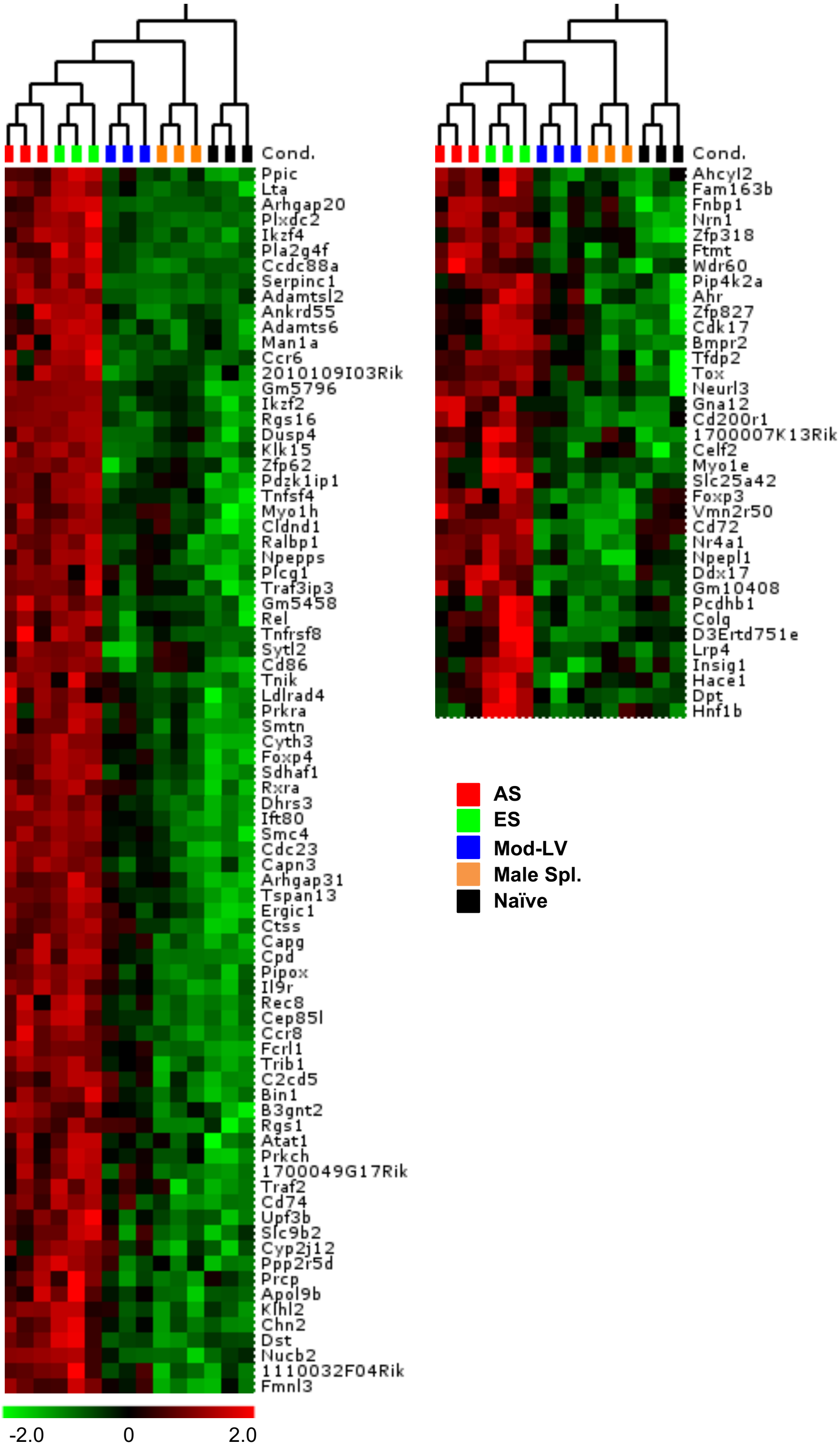

Supplementary Figure 3: List of the genes specifically upregulated in Marilyn cells activated in the tumor context. Heatmap and hierarchical clustering of the 116 genes defined in fig. 2a.

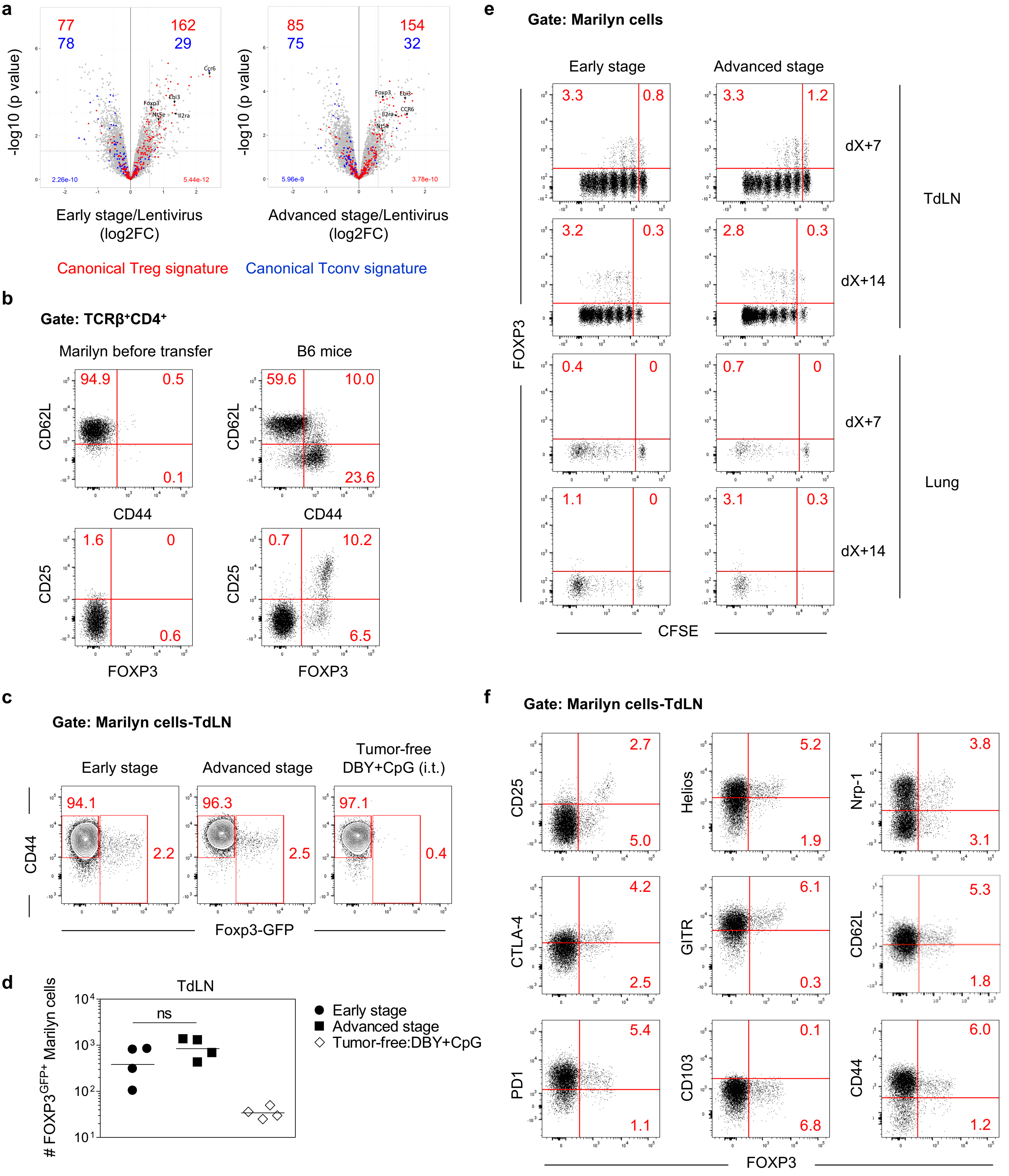

Supplementary Figure 4: Tumor Ag-specific pTregs are generated at early stage of tumor development. (a) Whole transcriptome comparison of ES vs LV and AS vs LV conditions (detailed in fig. 2a). Grey dots represent the fold change and p value between the indicated conditions for each gene. Red dots represent the canonical Treg signature; blue dots represent the canonical conventional T cell signature. Numbers correspond to the genes belonging to the Treg (red) or conventional T cell (blue) signature. The enrichment p value for each signature is shown. **(b)** Phenotype of Marilyn cells before adoptive transfer as compared to $CD4^+$ T cells from B6 control mice. **(c, d)** FACS-purified FOXP3-GFP^{Neg} naïve Marilyn cells were transferred into mice bearing ES or AS tumors or into tumor-free B6 mice injected with DBY+CpG i.t. **(c)** Representative plots of FOXP3-GFP^{pos} Marilyn cells and **(d)** Quantification (number). Representative of one out of two independent experiments. ns: non-significant unpaired t-test. **(e)** FOXP3 expression by undivided and divided Marilyn cells 7 and 14 days after adoptive transfer. **(f)** Expression of Treg-related markers by $FOXP3^+$ Marilyn cells 7 days after transfer into mice bearing advanced tumors. Representative of two independent experiments.

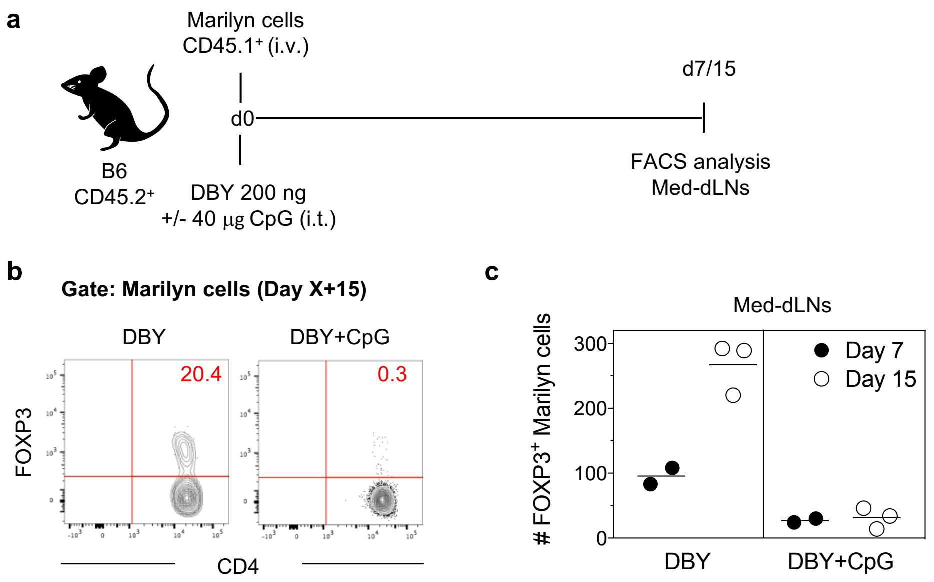

Supplementary Figure 5: CpG impairs pTregs generation induced by DBY peptide alone. (a) Tumor-free B6 mice harboring naïve Marilyn cells were administered with DBY peptide (200 ng) i.t. with or without CpG. **(b)** Representative plots showing the frequency of Marilyn cells expressing FOXP3 in the Med-dLNs and **(c)** quantification (number). Representative of three independent experiments.

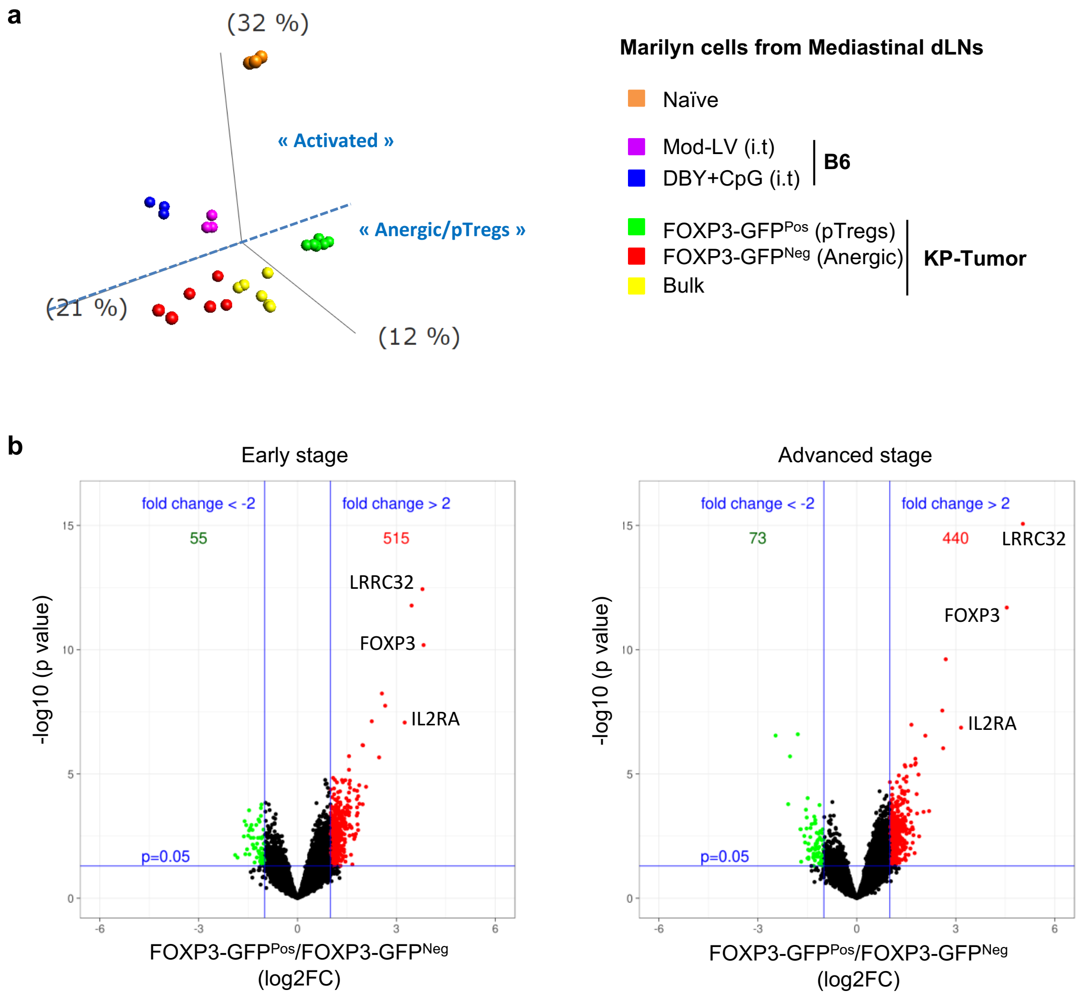

Supplementary Figure 6: Comparison of the transcriptomes of Marilyn pTregs and anergic cells. (a) Principal Component Analysis of bulk activated Marilyn cells and purified pTregs (FOXP-GFP^{Pos}) or anergic Marilyn cells (FOXP3-GFP^{Neg}) from TdLN of mice bearing early or advanced tumors 7 days after adoptive transfer. Naïve Marilyn cells or activated Marilyn cells from tumor-free mice administered with the Mod- LV or DBY+ CpG i.t. were used as controls ($q<0.05$). **(b)** Volcano plots representing the p value against fold-change gene expression for Marilyn pTregs versus anergic Marilyn cells from mice bearing early or advanced tumors. Upregulated or downregulated genes (Fold change>2, $p<0.05$) are highlighted in red and green, respectively.

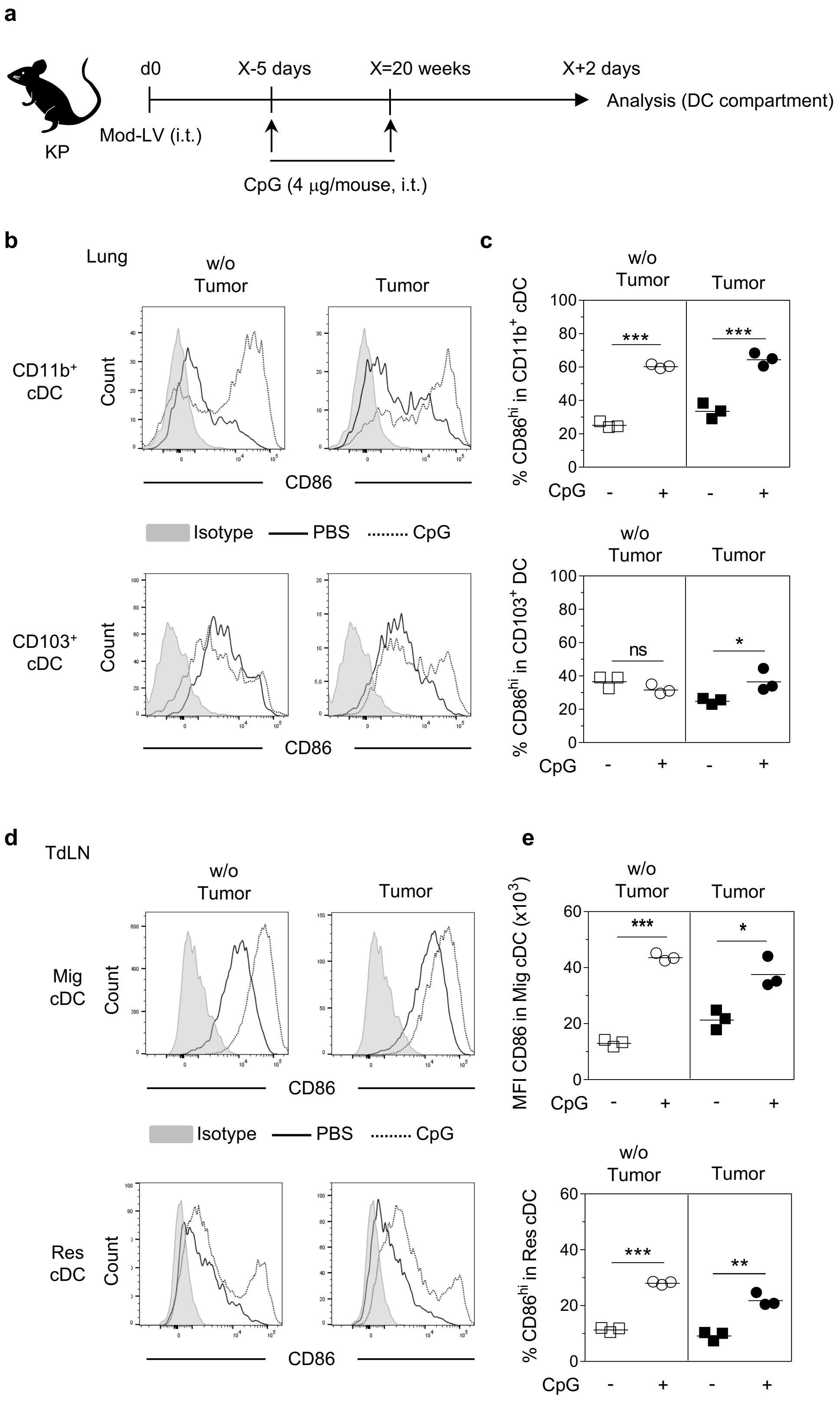

Supplementary Figure 7: CpG adjuvant efficiently promotes DC maturation both in the lung and in the TdLN. (a) Tumor-bearing or tumor-free B6 mice were injected i.t. twice with CpG (4 μg) at 5-day interval and DC compartment was analyzed 2 days later by FACS. **(b)** Representative pattern of CD86 expression in CD11b^{+} or CD103^{+} Lin^{-}(CD19^{-}TCRb^{-}TCRgamma/delta^{-}NK1.1^{-}) MHC-II^{hi}CD11c^{+} conventional DC (cDC) subsets from the lung of tumor-free or tumor-bearing mice receiving or not CpG and **(c)** quantification (frequency). **(d)** CD86 expression in migratory (MHC-II^{++}CCR7^{+}) or resident (MHC-II^{+}CCR7^{-}) cDC subsets in the Med-dLNs and **(e)** quantification (Mean Fluorescence Intensity (MFI) and frequency). One representative experiment out of two is shown. ns: non-significant, *p<0.05, **p<0.01, ***p<0.001 unpaired t-test.

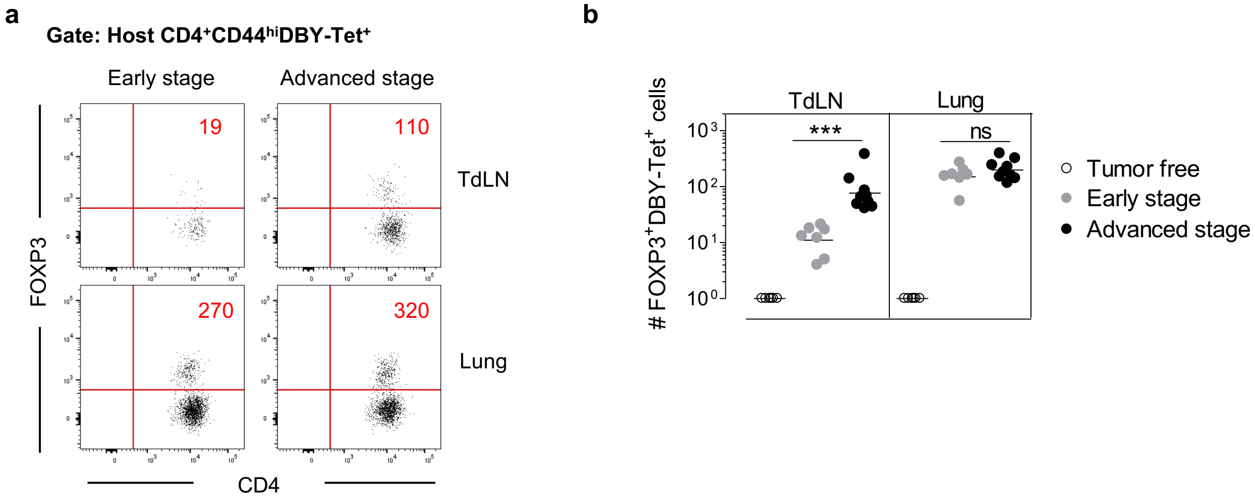

Supplementary Figure 8: Tumor Ag-specific host Tregs accumulate during tumor development (a) Representative plots of FOXP3 staining among CD44^{hi} host CD4^{+} T cells following DBY:I-A^{b} tetramer (Tet)-based cell enrichment of cell suspensions from TdLN and lung of mice bearing early or advanced tumors. Total numbers of DBY-specific host Tregs are shown. **(b)** Quantification (number). Pooled data of two independent experiments. ns: non-significant; ***p<0.001 Mann Whitney *U* test.

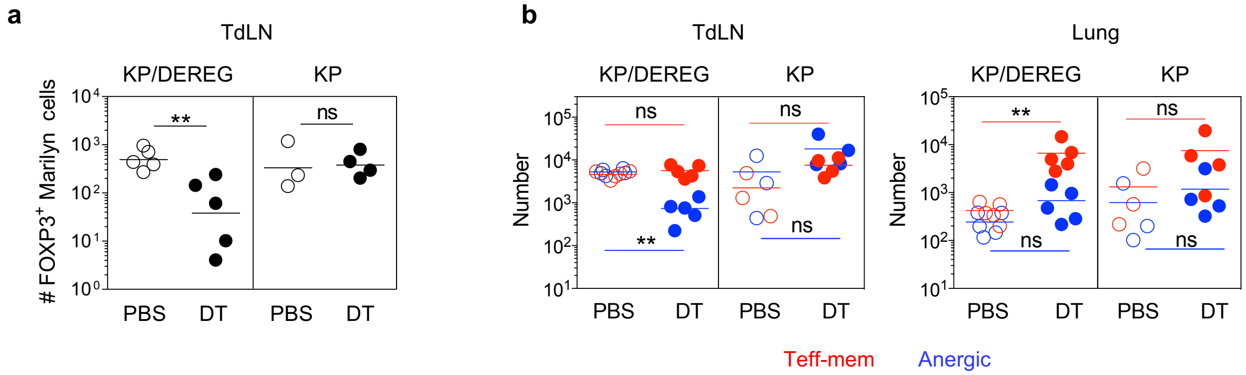

Supplementary Figure 9: The induction of pTregs and the acquisition of the anergic phenotype are not associated with DT-induced inflammation. Chimeric mice (KP/DEREG) or KP mice bearing tumors were treated with DT or PBS following the scheme defined in figure 8a. **(a)** Number of Marilyn cells expressing FOXP3 in the TdLN. **(b)** Number of Teff-mem or anergic Marilyn cells. ns: non-significant, ***p<0.001 Mann-Whitney *U* test.

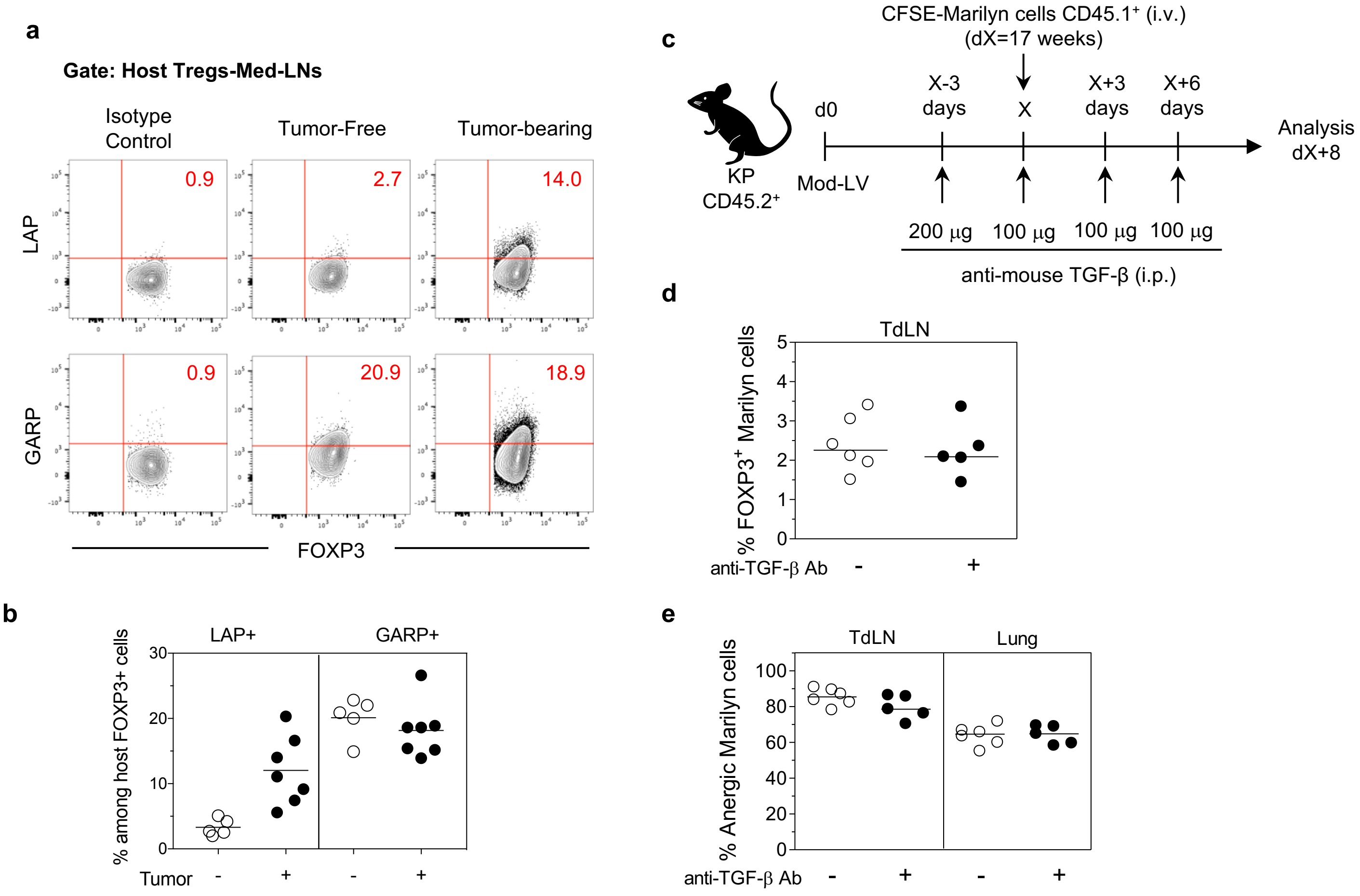

Supplementary Figure 10: TGF-β signaling is dispensable for the generation of tumor-specific Tregs and anergic cells. (a) Representative plots of LAP and GARP cell surface expression in total host Tregs from Med-LNs of tumor-free or tumor-bearing mice. **(b)** Quantification (frequency). **(c)** Experimental schedule of the anti-TGF-β treatment in tumor-bearing mice. Anti-mouse TGF-β neutralizing antibody was administered i.p. every 3 days in mice bearing tumors starting 3 days before Marilyn cell transfer. **(d)** Frequency of Marilyn pTregs and **(e)** frequency of anergic Marilyn cells in the TdLN and in the lung of tumor-bearing mice left untreated or treated with anti-TGF-β neutralizing antibody. One representative experiment out of two is depicted.

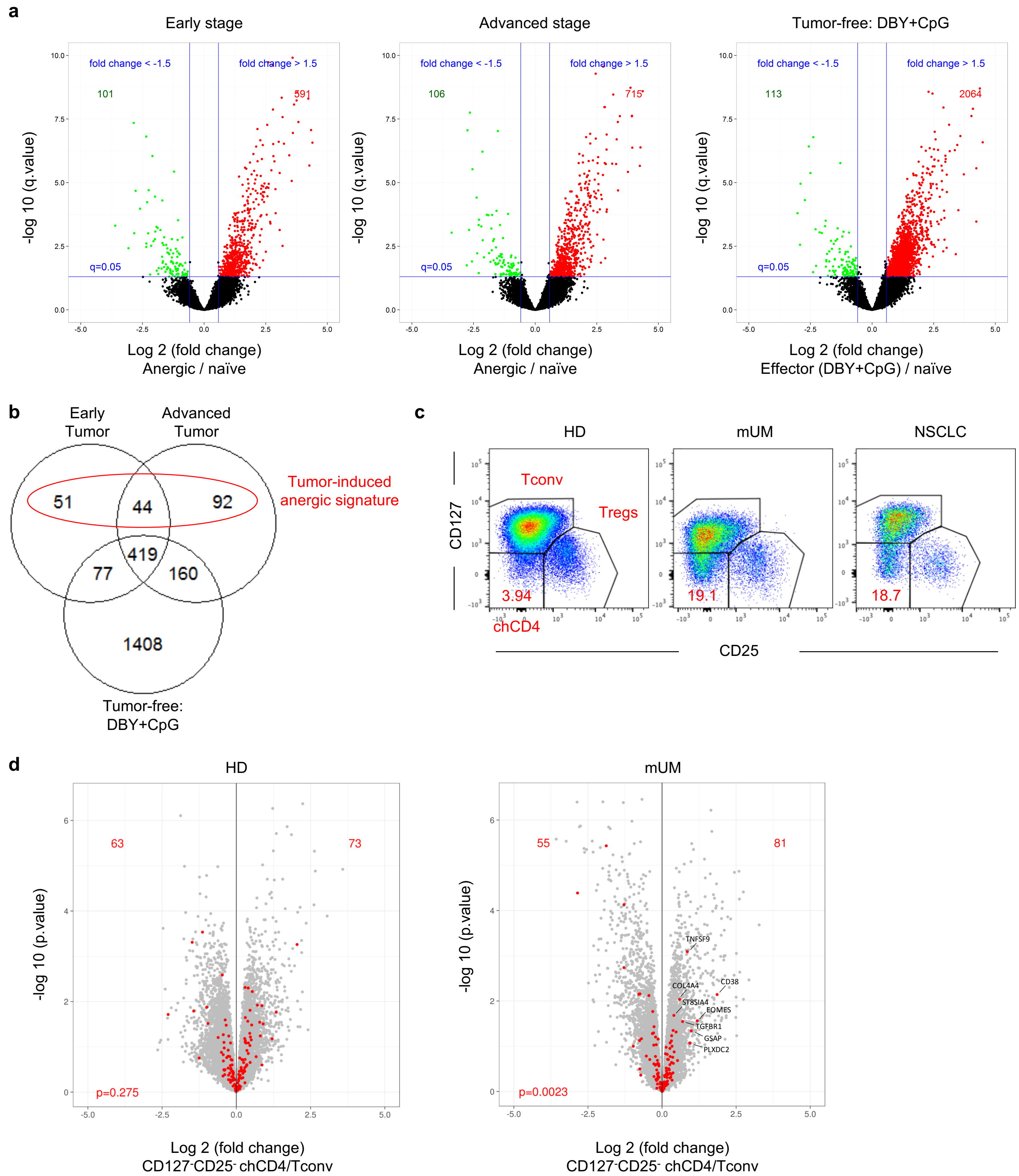

Supplementary Figure 11: Tumor-induced anergic signature is enriched in human cancer. (a) Volcano plots representing the q value against fold-change gene expression for FACS-purified anergic or effector Marilyn cells versus naïve Marilyn cells. Anergic Marilyn cells were purified from TdLN of mice bearing early or advanced tumors. Effector Marilyn cells were purified from tumor-free mice receiving DBY+CpG i.t. Upregulated or downregulated genes (Fold change>1.5, q<0.05) are highlighted in red and green, respectively. **(b)** Venn-diagram of genes upregulated in anergic and effector Marilyn cells. In red are depicted the 187 genes specifically upregulated in the anergic cells from tumor conditions representing the tumor-induced anergic signature. **(c)** Representative plots showing increased frequency of $CD127^{-}CD25^{-}CD4^{+}CD3^{+}$ T cells in the blood of patients with different types of cancer (healthy donor= HD, metastatic uveal melanoma=mUM, non-small cell lung cancer=NSCLC). The expression of CD127 and CD25 defines three subsets among $CD4^{+}$ T cells in human peripheral blood leucocytes (Tconv=$CD127^{+}CD25^{\pm}$ conventional $CD4^{+}$ T cells, Tregs=$CD127^{low}CD25^{+}$ cells, chCD4=$CD127^{-}CD25^{-}$ chronically activated $CD4^{+}$ T cells). **(d)** Volcano plots comparing the transcriptomes of chCD4 to the one of Tconv from healthy donors or metastatic uveal melanomas. In red is the anergic signature defined in **(b)**. The enrichment p value for each signature is shown.

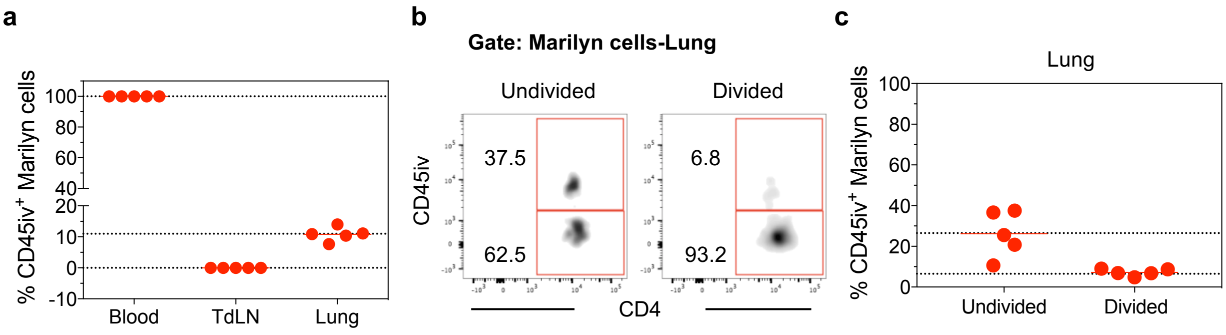

Supplementary Figure 12: Intravascular cells are removed from lung-perfused samples. Naïve Marilyn cells were transferred into tumor-bearing mice that received i.v. 3 µg of anti-CD45 antibody (CD45iv) 7 days later. Three minutes after injection, the lungs were perfused and harvested. **(a)** Frequency of circulatory CD45iv+ Marilyn cells in the blood (positive control), TdLN (negative control) and lung showing that only few residual blood cells are present in the perfused lung tissue. **(b)** Representative plots of CD45iv staining in undivided and divided lung Marilyn CD4^{+} T cells showing that residual blood cells represent a minor proportion of the undivided and divided compartment. **(c)** Quantification (frequency).
