# Supplementary material for: Induction of anergic or regulatory tumor-specific CD4+ T cells in the tumor-draining lymph node
Source: Nat Commun. 2018 May 29;9:2113. doi: 10.1038/s41467-018-04524-x (PMC5974295; doi:10.1038/s41467-018-04524-x)
Supplement: Supplementary file 3 — Description of Additional Supplementary Files [file 41467_2018_4524_MOESM3_ESM.pdf]

Description of Additional Supplementary Files

File Name: Supplementary Data 1
Description: Summary of pathological and immunohistochemical analyses. Eight animals were studied at each tumor stage. Detailed analysis of the TdLN was performed in 5 animals at each tumor stage. Quantitative results are expressed as mean ± SD

File Name: Supplementary Data 2
Description: Gene signature of tumor-induced Marilyn pTregs as compared to host nTregs and pTregs generated in tumor-free mice

File Name: Supplementary Data 3
Description: Gene signature of tumor-induced anergic Marilyn cells as compared to effector cells generated in tumor-free mice

File Name: Supplementary Data 4
Description: Summary of antibodies used in the study
